# Digital health literacy and infodemic awareness among preschool teachers: skills, behaviors, and determinants

**DOI:** 10.3389/fpubh.2026.1774132

**Published:** 2026-03-05

**Authors:** Aslihan Çelik Çoban, Yildiz Büyükdereli Atadag

**Affiliations:** Department of Family Medicine, Gaziantep University Medical Faculty, Gaziantep, Türkiye

**Keywords:** digital health, infodemic, literacy, preschool, teacher

## Abstract

**Objective:**

The aim of this study is to determine the levels of digital health literacy and infodemic awareness among preschool teachers, to examine the associated skills and behaviors, and to identify the influencing factors.

**Methods:**

This cross-sectional study was conducted between October–November 2025 among preschool teachers working in public schools in the province of Gaziantep. Data were collected through a questionnaire including socio-demographic characteristics, the Digital Health Literacy Instrument (DHLI), and the Infodemic Scale.

**Results:**

A total of 325 teachers aged between 19 and 59 participated in the study; 94.5% were female, and 66.2% were married. Daily internet/social media use was mostly 1–3 h (65.5%), and Instagram was the most frequently used platform (87.7%). Health information searching was reported as “sometimes/frequently” by 76.9%, with the main sources being Google (56%) and physicians (54.5%). The presence of chronic illness (self/family member) was 48.9%; in this group, both health information seeking and the use of Google and forums were higher. The mean DHLI score was 2.59 ± 0.38, and the Infodemic Scale score was 62.36 ± 6.35. Infodemic scores were higher among those who used the internet for 0–1 h and those who used television as a source of health information. DHLI predicted infodemic at a low but statistically significant level; in the multiple model, only “Adding Content” was significant.

**Conclusion:**

In the study, preschool teachers had a moderate level of digital health literacy, with differences among the subscales. “Adding Content” was stronger, while Protecting Privacy was weaker. Although digital health literacy was associated with infodemic, its effect was limited; in the multiple analysis, only “Adding Content” was significant. Training in verification, privacy, and evidence-based content, in collaboration with family physicians, may contribute to strengthening teachers' infodemic management and public health interventions.

## 1 Introduction

Preschool children (aged 3–6) are at high risk for anemia, obesity, emotional-behavioral problems, visual impairments, dental caries, and infectious diseases (1–7). To maintain a healthy life, individuals should be encouraged to take responsibility for their own health starting from early childhood (8). Preschool teachers play a strategic role in supporting individual and public health behaviors. These individuals are in a position to filter and share necessary health information not only for their own health but also for children, families, and colleagues (9).

Digital health literacy refers to the cognitive and social skills that enable individuals to access, understand, and use information for the purpose of protecting and promoting health through digital environments (such as websites, mobile applications, telehealth platforms, social media, artificial intelligence, etc.) (10). Supporting these skills in the early stages of life is important. Therefore, the development of health literacy among children and adolescents is generally carried out in collaboration with the education sector rather than the health sector (11). In this context, when teachers possess health literacy, they also gain the potential to effectively impart healthy living habits to children (9).

According to the World Health Organization, “infodemic” refers to the overabundance of both accurate and inaccurate information in digital and physical environments during epidemics, which causes confusion, increases risky behaviors, undermines trust in health authorities, and weakens public health interventions (12). Enhancing infodemic awareness and infodemic literacy within society is particularly important for teachers, who play a key role in information dissemination. Infodemic awareness is regarded as the ability to recognize misinformation and information overload, whereas infodemic literacy is defined as the capacity to critically evaluate, verify, and appropriately use health-related information within the context of an infodemic. It has been shown that individuals with low health literacy lack the ability to accurately evaluate online health information (13). One of the duties of primary healthcare services is to identify and guide the digital health literacy levels of key groups such as preschool teachers, who guide the public in information dissemination, within the scope of improving individuals' health literacy (14).

The aim of this study is to measure the digital health literacy levels and infodemic awareness of preschool teachers working in kindergartens, and to evaluate the relationship between these two variables. As a result of the study, it is expected that meaningful findings will be obtained regarding how teachers manage health information in digital environments, and that these findings will contribute to health literacy education, digital media use, and public health policies.

**Figure 1:**
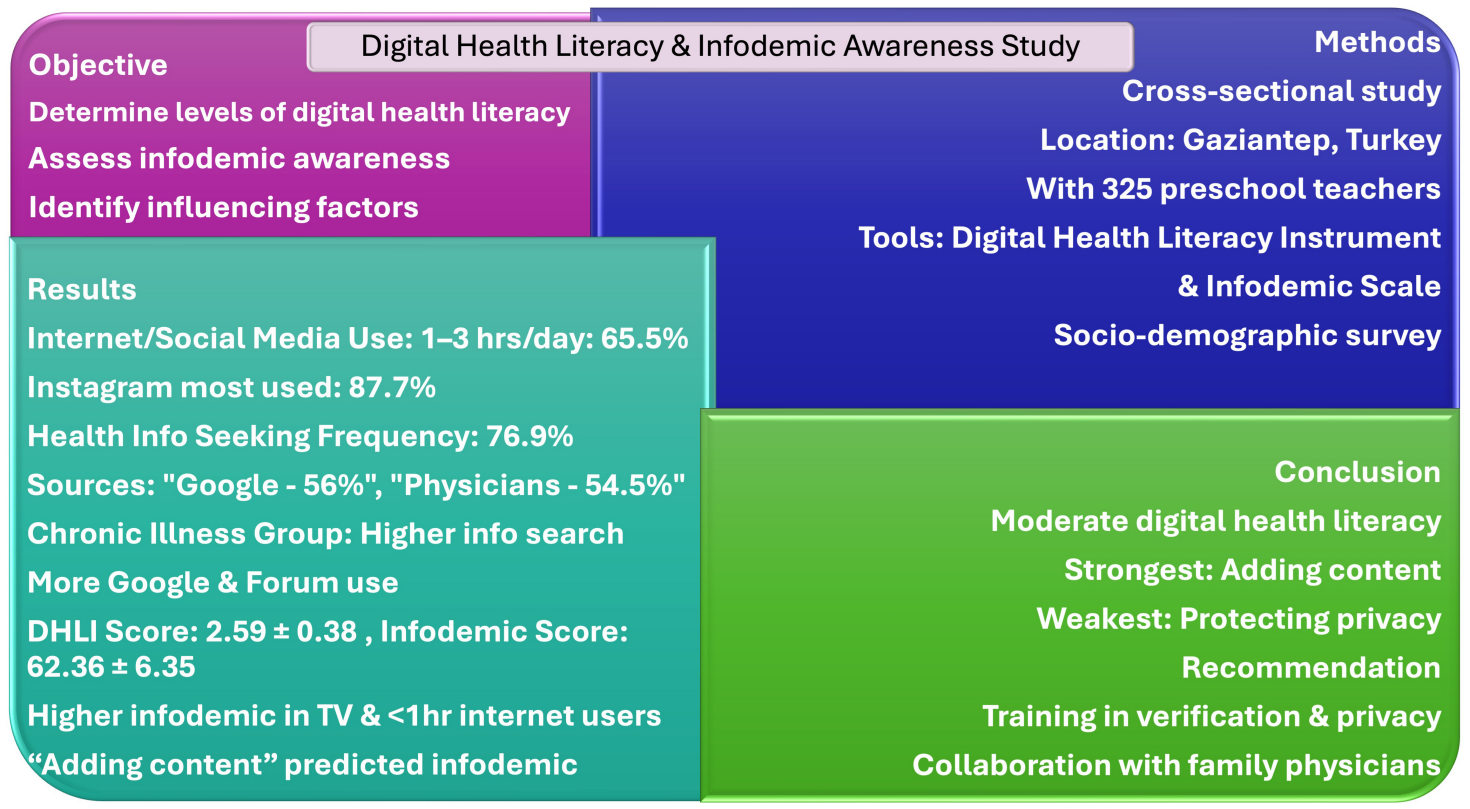
Graphical summary of the study design and main findings.

The overall design of the study and its main findings are summarized in Figure 1.

## 2 Methods

This study is cross-sectional and descriptive in nature. A questionnaire consisting of 42 questions was applied online to preschool teachers who voluntarily agreed to participate in the study.

### 2.1 Study participants

The study population consists of preschool teachers working in public schools in Gaziantep, Türkiye. The sample size was calculated as 310 based on a 95% confidence interval, 5% margin of error, and 50% response rate assumption. A total of 325 teachers who provided informed consent were included in the study.

Ethics committee approval was obtained from Gaziantep University (Decision No: 2025/243, Date: September 10, 2025), and permission to conduct the study was obtained from the Ministry of National Education (MEB.TT.2025.029961.01). The study was conducted between October 10 and November 10, 2025, in public schools affiliated with the Gaziantep Directorate of National Education.

### 2.2 Questionnaire

The questionnaire form, prepared following a literature review, was administered online via the Google Forms platform. The questionnaire consists of three sections and 42 questions. The first section of the questionnaire includes demographic information of the teachers (age, gender, marital status, years of teaching experience), duration of internet/social media use, commonly used platforms, frequency of searching for health information, preferences for health information, presence of health problems in the last year, and presence of chronic illness.

In the second section of the questionnaire, the Digital Health Literacy Instrument (DHLI) was used to measure teachers' skills in accessing, evaluating, and using health information in digital environments. The Turkish version of the Digital Health Literacy Instrument has been validated through exploratory and confirmatory factor analyses, and the overall Cronbach's alpha coefficient has been reported as 0.896. The instrument consists of 18 items and 6 sub-dimensions. These sub-dimensions are: information searching, evaluation of reliability, adding content, protection of privacy, determining relevance, and navigation skills. A 4-point Likert scale is used for the sub-dimensions of Information Searching, Evaluation of Reliability, Determining Relevance, and Adding Content (4 = Very easy, 3 = Easy, 2 = Difficult, 1 = Very difficult). For the sub-dimensions of Navigation Skills and Protection of Privacy, a reverse-coded 4-point Likert scale is used (Never = 4, Rarely = 3, Occasionally = 2, Frequently = 1). The scores obtained from the scale are interpreted as means: below 2 is considered low, between 2 and 3 is moderate, and above 3 is considered high digital health literacy (15, 16).

In the third section of the questionnaire, the Infodemic Scale was used to assess teachers' approaches to infodemic (17). The Infodemic Scale consists of 14 items and two sub-dimensions (awareness and literacy). The Awareness sub-dimension consists of 9 items [items 1–2–3–4–5–6–7–8–9], and the Literacy sub-dimension consists of 5 items [items 10–11–12–13–14]. The Infodemic Scale is a 5-point Likert scale (1 = Strongly disagree, 2 = Disagree, 3 = Neutral, 4 = Agree, 5 = Strongly agree). There are no reverse-coded items. The total score of the scale and the scores of its sub-dimensions are calculated by summing the item scores. The minimum score is 14 and the maximum is 70. Higher scores indicate a higher level of awareness and literacy regarding infodemic. The Cronbach's alpha value is 0.87 for the total scale, 0.77 for the Awareness sub-dimension, and 0.88 for the Literacy sub-dimension (17).

### 2.3 Statistical analysis

Data were analyzed using IBM SPSS Statistics (version 26.0). Descriptive statistics were presented using numbers, percentages, means, and standard deviations, while relationships between categorical variables were assessed with the chi-square test. Whether numerical variables conformed to normal distribution was analyzed using the Kolmogorov-Smirnov and Shapiro-Wilk tests. For numerical variables not conforming to normal distribution, the Mann-Whitney U-test and Kruskal-Wallis test were used. Relationships between variables, when normal distribution was achieved, were evaluated using Pearson correlation coefficient. A *p-value* less than 0.05 was considered statistically significant in all analyses.

## 3 Results

### 3.1 Sociodemographic data

A total of 325 preschool teachers participated in our study. Of the teachers, 307 (94.5%) were female and 18 (5.5%) were male. The mean age was 35.14 ± 8.11 years (min = 19, max = 59). Of the participants, 215 (66.2%) were married. The average professional experience of the teachers was 10.98 ± 7.61 years. In terms of experience, 29.5% had 1–4 years, 22.2% had 5–10 years, 21.2% had 11–16 years, and 27.1% had 17 years or more. Sociodemographic data of the participants are presented in Table 1.

**Table 1:** Descriptive characteristics of the participants.

**Variable**	**Category**	***n***	**%**
Age	≤ 29 years	97	29.8
	30–39 years	118	36.3
	≥40 years	110	33.8
Gender	Female	307	94.5
	Male	18	5.5
Marital status	Married	215	66.2
	Single	110	33.8
Duration of professional experience	1–4 years	96	29.5
	5–10 years	72	22.2
	11–16 years	69	21.2
	≥17 years	88	27.1
Daily internet/social media usage time	0–1 h	52	16.0
	1–3 h	213	65.5
	≥4 h	60	18.5
Most frequently used social media platforms^*^	Instagram	285	87.7
	Facebook	19	5.8
	Twitter (X)	58	18.0
	TikTok	4	1.2
	YouTube	131	40.3
	WhatsApp	243	74.8
	Other	19	5.8
Frequency of searching for health information in digital environments	Never	11	3.4
	Rarely	64	19.7
	Sometimes	143	44.0
	Frequently	107	32.9
Sources of health information in digital environments^*^	Ministry of health	124	38.2
	Physicians	177	54.5
	Forums	38	11.7
	Social media posts	67	20.6
	Google	182	56.0
	Television	16	4.9
	Artificial intelligence programs (such as ChatGPT, Gemini, Grok)	91	28.0
	Other	9	2.8
Presence of a serious diagnosis or treatment process within the last year	Yes	42	12.9
	No	283	87.1
Presence of a chronic disease in the individual or a first-degree relative	Yes	159	48.9
	No	166	51.1

^*^Multiple responses allowed.

When participants' daily internet/social media use was examined, 16% used it for 0–1 h, 65.5% for 1–3 h, and 18.5% for 4 h or more. The most frequently used social media platforms were Instagram (87.7%), WhatsApp (74.8%), YouTube (40.3%), Twitter/X (18.0%), TikTok (1.2%), and Facebook (5.8%). In terms of frequency of health information seeking, 32.9% reported “frequently,” 44% “sometimes,” 19.7% “rarely,” and 3.4% “never.” The digital health information sources used by participants and their respective rates were: Google (56%), physicians (54.5%), Ministry of Health (38.2%), artificial intelligence programs (28%), social media posts (20.6%), TV programs (4.9%), and other (2.8%). It was determined that 12.9% of the teachers had experienced a serious diagnosis or treatment process in the past year. Additionally, 48.9% reported having a chronic illness either personally or in a first-degree relative (Table 1).

Teachers with a personal or first-degree relative's chronic illness searched for health information on the internet significantly more frequently (*p* = 0.009). They also used Google and forums significantly more often as digital health information sources (*p* = 0.008, *p* = 0.027).

### 3.2 Digital health literacy scale score

The Cronbach's alpha values for the subscales of the DHLI ranged from 0.745 to 0.916, and the total scale score was calculated as 0.798. The teachers' average total DHLI score was 2.59 ± 0.38 (Min = 1.44, Max = 4). Subscale means were: Information Searching 2.90 ± 0.59, Evaluation of Reliability 2.61 ± 0.68, Determining Relevance 2.85 ± 0.65, Navigation Skills 2.25 ± 0.77, Adding Content 3.15 ± 0.69, and Protecting Privacy 1.78 ± 0.72.

When DHLI and its subscale scores were compared across age groups, gender, marital status, and years of professional experience, no statistically significant differences were found (*p* > 0.05). These comparisons are shown in Tables 2, 3.

**Table 2:** DHLI scores by age, gender, and marital status.

**Sociodemographic variable**	**Scale/Subscale**	**Group**	***n***	**Mean score**	**SD**	***p***
Age	Information searching	<30 years	97	2.90	0.58	0.995
		30–39 years	118	2.90	0.54	
		≥40 years	110	2.90	0.63	
	Evaluating reliability	<30 years	97	2.66	0.70	0.382
		30–39 years	118	2.63	0.59	
		≥40 years	110	2.54	0.73	
	Determining relevance	<30 years	97	2.95	0.62	0.110
		30–39 years	118	2.85	0.62	
		≥40 years	110	2.76	0.70	
	Navigation skills	<30 years	97	2.23	0.88	0.817
		30–39 years	118	2.29	0.72	
		≥40 years	110	2.23	0.72	
	Adding content	<30 years	97	3.23	0.73	0.378
		30–39 years	118	3.10	0.67	
		≥40 years	110	3.14	0.69	
	Protecting privacy	<30 years	97	1.84	0.76	0.519
		30–39 years	118	1.78	0.68	
		≥40 years	110	1.72	0.71	
	Total scale score	<30 years	97	2.64	0.39	0.249
		30–39 years	118	2.59	0.36	
		≥40 years	110	2.55	0.39	
Gender	Information searching	Female	307	2.89	0.58	0.194
		Male	18	3.07	0.61	
	Evaluating reliability	Female	307	2.59	0.67	0.052
		Male	18	2.91	0.67	
	Determining relevance	Female	307	2.84	0.65	0.310
		Male	18	3.00	0.70	
	Navigation skills	Female	307	2.24	0.75	0.472
		Male	18	2.43	1.05	
	Adding content	Female	307	3.14	0.68	0.312
		Male	18	3.31	0.85	
	Protecting privacy	Female	307	1.76	0.68	0.334
		Male	18	2.04	1.15	
	Total scale score	Female	307	2.58	0.36	0.162
		Male	18	2.79	0.62	
Marital status	Information searching	Married	215	2.92	0.59	0.393
		Single	110	2.86	0.58	
	Evaluating reliability	Married	215	2.62	0.67	0.629
		Single	110	2.58	0.68	
	Determining relevance	Married	215	2.84	0.64	0.853
		Single	110	2.86	0.67	
	Navigation skills	Married	215	2.24	0.73	0.694
		Single	110	2.28	0.83	
	Adding content	Married	215	3.14	0.70	0.604
		Single	110	3.18	0.68	
	Protecting privacy	Married	215	1.76	0.71	0.453
		Single	110	1.82	0.72	
	Total scale score	Married	215	2.59	0.38	0.829
		Single	110	2.60	0.38	

**Table 3:** DHLI scores by professional service and daily internet/social media use time.

**Sociodemographic variable**	**Scale/Subscale**	**Group**	***n***	**Mean score**	**SD**	***p***
Years of professional service	Information searching	<5 years	96	2.95	0.60	0.284
		5–10 years	72	2.88	0.53	
		11–16 years	69	2.79	0.54	
		≥17 years	88	2.95	0.64	
	Evaluating reliability	<5 years	96	2.72	0.68	0.185
		5–10 years	72	2.63	0.65	
		11–16 years	69	2.51	0.61	
		≥17 years	88	2.55	0.74	
	Determining relevance	<5 years	96	2.94	0.63	0.130
		5–10 years	72	2.91	0.64	
		11–16 years	69	2.73	0.66	
		≥17 years	88	2.78	0.67	
	Navigation skills	<5 years	96	2.20	0.84	0.673
		5–10 years	72	2.27	0.76	
		11–16 years	69	2.34	0.73	
		≥17 years	88	2.22	0.72	
	Adding content	<5 years	96	3.26	0.61	0.124
		5–10 years	72	3.18	0.77	
		11–16 years	69	3.00	0.72	
		≥17 years	88	3.13	0.68	
	Protecting privacy	<5 years	96	1.89	0.79	0.227
		5–10 years	72	1.69	0.71	
		11–16 years	69	1.80	0.56	
		≥17 years	88	1.71	0.75	
	Total scale score	<5 years	96	2.66	0.39	0.123
		5–10 years	72	2.59	0.36	
		11–16 years	69	2.53	0.34	
		≥17 years	88	2.56	0.40	
Daily internet/social media usage time	Information searching	0–1 h	52	2.96	0.68	0.254
		1–3 h	213	2.86	0.57	
		≥4 h	60	2.98	0.56	
	Evaluating reliability	0–1 h	52	2.62	0.84	0.955
		1–3 h	213	2.60	0.64	
		≥4 h	60	2.63	0.64	
	Determining relevance	0–1 h	52	2.83	0.78	0.579
		1–3 h	213	2.83	0.61	
		≥4 h	60	2.93	0.68	
	Navigation skills	0–1 h	52	2.20	0.75	0.750
		1–3 h	213	2.28	0.75	
		≥4 h	60	2.22	0.83	
	Adding content	0–1 h	52	3.17	0.76	0.802
		1–3 h	213	3.14	0.61	
		≥4 h	60	3.20	0.89	
	Protecting privacy	0–1 h	52	1.54	0.59	**0.017**
		1–3 h	213	1.85	0.75	
		≥4 h	60	1.74	0.64	
	Total scale score	0–1 h	52	2.55	0.41	0.675
		1–3 h	213	2.59	0.39	
		≥4 h	60	2.62	0.33	

Bold values indicate statistical significance (*p* < 0.05).

According to daily internet/social media use duration, only the Protecting Privacy subscale showed a significant difference (*F* = 4.154, *p* = 0.017; η^2^ = 0.025), with the highest mean in the 1–3 h group (1.85 ± 0.75). Tukey's test showed the difference was between the 0–1 h and 1–3 h groups (Table 3).

No statistically significant difference was found between frequency of health information seeking and DHLI or its subscales (*p* > 0.05). Likewise, no significant difference was found in DHLI scores based on having experienced a serious diagnosis/treatment in the past year or having a chronic illness (*p* > 0.05). These comparisons are shown in Table 4.

**Table 4:** DHLI scores by health information–seeking frequency and health status.

**Sociodemographic variable**	**Scale/Subscale**	**Group**	***n***	**Mean score**	**SD**	***p***
Frequency of searching for health information on the internet	Information searching	Never	11	2.55	0.73	0.103
		Rarely	64	2.98	0.57	
		Sometimes	143	2.86	0.55	
		Frequently	107	2.93	0.61	
	Evaluating reliability	Never	11	2.15	0.89	0.075
		Rarely	64	2.67	0.66	
		Sometimes	143	2.57	0.64	
		Frequently	107	2.67	0.70	
	Determining relevance	Never	11	2.48	0.81	0.205
		Rarely	64	2.79	0.68	
		Sometimes	143	2.88	0.60	
		Frequently	107	2.88	0.68	
	Navigation skills	Never	11	2.15	0.69	0.734
		Rarely	64	2.17	0.81	
		Sometimes	143	2.28	0.74	
		Frequently	107	2.28	0.78	
	Adding content	Never	11	3.21	0.85	0.887
		Rarely	64	3.09	0.73	
		Sometimes	143	3.17	0.64	
		Frequently	107	3.17	0.73	
	Protecting privacy	Never	11	1.82	0.38	0.415
		Rarely	64	1.69	0.66	
		Sometimes	143	1.85	0.77	
		Frequently	107	1.73	0.70	
	Total scale score	Never	11	2.39	0.40	0.311
		Rarely	64	2.57	0.30	
		Sometimes	143	2.60	0.40	
		Frequently	107	2.61	0.39	
Presence of a serious diagnosis or treatment process within the last year	Information searching	Yes	42	2.83	0.62	0.381
		No	283	2.91	0.58	
	Evaluating reliability	Yes	42	2.57	0.76	0.714
		No	283	2.61	0.66	
	Determining relevance	Yes	42	2.71	0.67	0.154
		No	283	2.87	0.65	
	Navigation skills	Yes	42	2.43	0.80	0.110
		No	283	2.23	0.76	
	Adding content	Yes	42	3.01	0.74	0.144
		No	283	3.18	0.69	
	Protecting privacy	Yes	42	1.85	0.76	0.499
		No	283	1.77	0.71	
	Total scale score	Yes	42	2.57	0.39	0.662
		No	283	2.59	0.38	
Presence of a chronic disease in the individual or a first-degree relative	Information searching	Yes	159	2.87	0.61	0.343
		No	166	2.93	0.56	
	Evaluating reliability	Yes	159	2.57	0.69	0.283
		No	166	2.65	0.66	
	Determining relevance	Yes	159	2.86	0.63	0.717
		No	166	2.84	0.67	
	Navigation skills	Yes	159	2.28	0.77	0.480
		No	166	2.22	0.77	
	Adding content	Yes	159	3.16	0.70	0.974
		No	166	3.15	0.69	
	Protecting privacy	Yes	159	1.81	0.69	0.496
		No	166	1.75	0.74	
	Total scale score	Yes	159	2.59	0.35	0.998
		No	166	2.59	0.41	

### 3.3 Infodemic scale score

For this sample, the Cronbach's alpha values were 0.839 for Awareness, 0.921 for Literacy, and 0.908 for the total scale. The mean total Infodemic Scale score was 62.36 ± 6.35 (Min = 38, Max = 70). The subscale means were: Awareness 40.24 ± 4.03 (Min = 27, Max = 45) and Literacy 22.12 ± 2.91 (Min = 9, Max = 25).

No statistically significant differences were found between Infodemic Scale scores (total or subscales) and age, gender, marital status, or years of experience (*p* > 0.05). These results are shown in Table 5.

**Table 5:** Infodemic scale scores by sociodemographic characteristics.

**Sociodemographic variable**	**Scale/Subscale**	**Group**	***n***	**Mean score**	**SD**	***p***
Age	Awareness	< 30 years	97	40.02	4.48	0.720
		30–39 years	118	40.21	3.95	
		≥40 years	110	40.47	3.70	
	Literacy	< 30 years	97	21.75	3.22	0.274
		30–39 years	118	22.16	2.56	
		≥40 years	110	22.40	2.96	
	Total scale score	< 30 years	97	61.77	7.09	0.463
		30–39 years	118	62.37	5.93	
		≥40 years	110	62.87	6.12	
Gender	Awareness	Female	307	40.17	4.05	0.174
		Male	18	41.50	3.55	
	Literacy	Female	307	22.06	2.93	0.098
		Male	18	23.22	2.21	
	Total scale score	Female	307	62.22	6.39	0.105
		Male	18	64.72	5.31	
Marital status	Awareness	Married	215	40.35	3.76	0.517
		Single	110	40.03	4.53	
	Literacy	Married	215	22.21	2.77	0.439
		Single	110	21.95	3.15	
	Total scale score	Married	215	62.56	6.05	0.429
		Single	110	61.97	6.91	
Years of professional service	Awareness	< 5 years	96	40.00	4.51	0.436
		5–10 years	72	40.13	4.03	
		11–16 years	69	39.94	3.72	
		≥17 years	88	40.84	3.70	
	Literacy	< 5 years	96	21.94	3.05	0.554
		5–10 years	72	22.00	2.85	
		11–16 years	69	22.01	2.78	
		≥17 years	88	22.50	2.90	
	Total scale score	< 5 years	96	61.94	6.95	0.409
		5–10 years	72	62.13	6.31	
		11–16 years	69	61.96	5.92	
		≥17 years	88	63.34	6.03	

When examined by daily internet/social media use, participants using the internet for 0–1 h had significantly higher Infodemic and subscale scores. Awareness scores were highest in this group (41.67 ± 3.45) compared to 1–3 h (39.97 ± 4.04) and 4+ h (39.97 ± 4.27). The difference was significant (*F* = 3.969, *p* = 0.020; η^2^ = 0.024), with Tukey's test identifying the difference between 0–1 and 1–3 h groups. Literacy scores were also highest in the 0–1 h group (23.04 ± 2.54), compared to 1–3 h (21.91 ± 2.89) and 4+ h (22.08 ± 3.13). The ANOVA was significant (*F* = 3.225, *p* = 0.041; η^2^ = 0.020). Infodemic total scores were also highest in the 0–1 h group (64.71 ± 5.70), compared to 1–3 h (61.88 ± 6.18) and 4+ h (62.05 ± 7.12) (*F* = 4.333, *p* = 0.014; η^2^ = 0.026). Tukey's test showed a significant difference between 0–1 and 1–3 h groups (Table 6).

**Table 6:** Infodemic scale scores by internet use and health status.

**Sociodemographic variable**	**Scale/Subscale**	**Group**	***n***	**Mean score**	**SD**	***p***
Daily internet/social media usage time	Awareness	0–1 h	52	41.67	3.45	**0.020**
		1–3 h	213	39.97	4.04	
		≥4 h	60	39.97	4.27	
	Literacy	0–1 h	52	23.04	2.54	**0.041**
		1–3 h	213	21.91	2.89	
		≥4 h	60	22.08	3.13	
	Total scale score	0–1 h	52	64.71	5.70	**0.014**
		1–3 h	213	61.88	6.18	
		≥4 h	60	62.05	7.12	
Frequency of searching for health information on the internet	Awareness	Never	11	43.09	2.59	**0.029**
		Rarely	64	39.95	3.69	
		Sometimes	143	39.80	4.18	
		Frequently	107	40.71	4.02	
	Literacy	Never	11	22.27	3.13	0.938
		Rarely	64	22.13	2.50	
		Sometimes	143	22.01	2.85	
		Frequently	107	22.24	3.20	
	Total scale score	Never	11	65.36	4.50	0.208
		Rarely	64	62.08	5.64	
		Sometimes	143	61.82	6.48	
		Frequently	107	62.95	6.68	
Presence of a serious diagnosis or treatment process within the last year	Awareness	Yes	42	40.36	3.45	0.845
		No	283	40.23	4.11	
	Literacy	Yes	42	21.57	3.35	0.190
		No	283	22.20	2.83	
	Total scale score	Yes	42	61.93	6.34	0.636
		No	283	62.43	6.37	
Presence of a chronic disease in the individual or a first-degree relative	Awareness	Yes	159	40.28	3.99	0.861
		No	166	40.20	4.08	
	Literacy	Yes	159	22.12	2.95	0.998
		No	166	22.12	2.87	
	Total scale score	Yes	159	62.40	6.27	0.913
		No	166	62.33	6.45	

Bold values indicate statistical significance (*p* < 0.05).

In terms of health information seeking frequency, only the Awareness subscale showed a statistically significant difference (*F* = 3.043, *p* = 0.029; η^2^ = 0.028). Teachers who never searched for online health information had the highest Awareness score (43.09 ± 2.59), followed by those who searched rarely (39.95 ± 3.69), sometimes (39.80 ± 4.18), and frequently (40.71 ± 4.02) (Table 6).

No significant differences were found in Infodemic Scale scores based on having had a serious diagnosis/treatment in the past year or having a chronic illness (*p* > 0.05; Table 6).

### 3.4 Correlation between scales

Correlation analysis showed very strong positive relationships among awareness, literacy, and total infodemic scores, especially between awareness-infodemic (*r* = 0.94, *p* < 0.001) and literacy-infodemic (*r* = 0.88, *p* < 0.001).

A simple linear regression analysis was conducted to determine the extent to which digital health literacy predicts infodemic level. The model was significant (*F* = 15.092, *p* < 0.001). The dependent variable was the infodemic score, and the independent variable was digital health literacy score. Although the explained variance (*R*^2^ = 0.045) was low, it was significant, indicating that digital health literacy explained approximately 4.5% of the variance in infodemic level. The regression coefficients showed that digital health literacy was a significant and positive predictor of infodemic (*B* = 3.531; SE = 0.909; β = 0.211; *t* = 3.885; *p* < 0.001), indicating that higher digital health literacy is associated with higher infodemic scores among teachers.

A multiple linear regression was performed to determine how well the subscales of digital health literacy collectively predicted infodemic level. The model was significant (*F* = 5.008, *p* < 0.001) with an explained variance of *R*^2^ = 0.086, indicating that the subscales accounted for 8.6% of the variance in infodemic scores. Among the subscales, only Adding Content was a significant predictor (*B* = 1.710; SE = 0.544; β = 0.187; *t* = 3.142; *p* = 0.002). The other subscales-Information Searching, Evaluation of Reliability, Determining Relevance, Navigation Skills, and Protecting Privacy-were not statistically significant predictors (*p* > 0.05; Table 7).

**Table 7:** Correlation matrix between scales.

**Variable**	**Statistic**	**Awareness**	**Literacy**	**Infodemic scale**	**Information searching**	**Evaluating reliability**	**Determining relevance**	**Navigation skills**	**Adding content**	**Protecting privacy**	**Digital health literacy scale**
Awareness	r	1									
	p										
Literacy	r	0.670^**^	1								
	p	0.000									
Infodemic scale	r	0.941^**^	0.882^**^	1							
	p	0.000	0.000								
Informations searching	r	0.177^**^	0.209^**^	0.208^**^	1						
	p	0.001	0.000	0.000							
Evaluating reliability	r	0.139^*^	0.162^**^	0.162^**^	0.707^**^	1					
	p	0.012	0.003	0.003	0.000						
Determining relevance	r	0.164^**^	0.185^**^	0.189^**^	0.646^**^	0.701^**^	1				
	p	0.003	0.001	0.001	0.000	0.000					
Navigation skills	r	−0.054	−0.113^*^	−0.086	−0.144^**^	−0.085	−0.123^*^	1			
	p	0.335	0.042	0.123	0.009	0.127	0.027				
Adding content	r	0.203^**^	0.244^**^	0.240^**^	0.320^**^	0.276^**^	0.379^**^	−0.224^**^	1		
	p	0.000	0.000	0.000	0.000	0.000	0.000	0.000			
Protecting privacy	r	0.061	−0.004	0.037	−0.006	0.065	−0.038	0.512^**^	−0.141^*^	1	
	p	0.275	0.949	0.507	0.920	0.243	0.493	0.000	0.011		
Digital health literacy scale	r	0.197^**^	0.190^**^	0.211^**^	0.698^**^	0.754^**^	0.721^**^	0.331^**^	0.457^**^	0.450^**^	1
	p	0.000	0.001	0.000	0.000	0.000	0.000	0.000	0.000	0.000	

r, Pearson correlation coefficient. ^*^p < 0.05, ^**^p < 0.01.

In multivariate regression models, Adding Content remained the only significant predictor of infodemic, awareness, and literacy scores when all digital health literacy subscales were included.

### 3.5 Scale scores by health information source

The teachers' digital health information sources were compared with their scale scores. Infodemic scores of those who used TV as a source were significantly higher [Median (IQR) = 67.5 (5.75)] compared to non-users [Median (IQR) = 63.0 (12.0)] (Mann-Whitney U = 1,612.5, *p* = 0.018, *r* = 0.13). Additionally, those who used “Other” digital sources had significantly higher DHLI scores [Median (IQR) = 2.64 (0.63)] than non-users [Median (IQR) = 2.55 (0.44)] (Mann-Whitney U = 662.0, *p* = 0.006; Table 8).

**Table 8:** Comparison of infodemic scale and digital health literacy scale scores according to digital health information source selection.

**Information source**	**Scale**	**Group**	***n***	**Median (IQR)**	**Mann–Whitney U**	***p***	**Effect size (r)**
Ministry of Health	Infodemic Scale	Yes	124	64.0 (11.0)	11,250.5	0.139	0.08
		No	201	63.0 (12.0)			
	Digital Health Literacy Scale	Yes	124	2.61 (0.39)	11,569.0	0.277	0.06
		No	201	2.56 (0.44)			
Physicians	Infodemic Scale	Yes	177	64.0 (10.0)	11,754.5	0.110	0.09
		No	148	62.0 (12.0)			
	Digital Health Literacy Scale	Yes	177	2.61 (0.42)	11,956.5	0.175	0.08
		No	148	2.50 (0.43)			
Forums	Infodemic Scale	Yes	38	64.5 (11.25)	4,644.5	0.136	0.08
		No	287	63.0 (12.0)			
	Digital Health Literacy Scale	Yes	38	2.64 (0.63)	5,253.5	0.713	0.02
		No	287	2.55 (0.44)			
Social media posts	Infodemic Scale	Yes	67	62.0 (11.0)	7,993.5	0.341	0.05
		No	258	64.0 (12.0)			
	Digital Health Literacy Scale	Yes	67	2.61 (0.44)	7,796.5	0.216	0.07
		No	258	2.55 (0.44)			
Google	Infodemic Scale	Yes	182	63.5 (12.0)	12,074.0	0.262	0.06
		No	143	63.0 (11.0)			
	Digital Health Literacy Scale	Yes	182	2.55 (0.46)	12,737.0	0.742	0.02
		No	143	2.55 (0.44)			
Television	Infodemic Scale	Yes	16	67.5 (5.75)	1,612.5	**0.018**	0.13
		No	309	63.0 (12.0)			
	Digital Health Literacy Scale	Yes	16	2.69 (0.49)	1,995.0	0.192	0.07
		No	309	2.55 (0.44)			
Artificial intelligence programs (such as ChatGPT, Gemini, Grok)	Infodemic Scale	Yes	91	62.0 (13.0)	10,550.0	0.898	0.05
		No	234	64.0 (11.0)			
	Digital Health Literacy Scale	Yes	91	2.55 (0.44)	9,962.5	0.367	0.05
		No	234	2.61 (0.40)			
Other	Infodemic Scale	Yes	9	64.0 (11.25)	1,187.5	0.397	0.01
		No	316	63.0 (12.0)			
	Digital Health Literacy Scale	Yes	9	2.44 (0.33)	662.0	**0.006**	0.15
		No	316	2.55 (0.44)			

Bold values indicate statistical significance (*p* < 0.05).

## 4 Discussion

In this study, the digital health literacy (DHL) and infodemic awareness of preschool teachers were examined together. The findings revealed differences among the subscales of digital health literacy and indicated that particularly the skill of adding content in digital environments might be a predictor of the level of infodemic awareness.

When asked about the frequency of searching for health information, 32.9% of the teachers responded “frequently,” 44% “sometimes,” 19.7% “rarely,” and 3.4% “never.” This distribution suggests that health information-seeking behavior is generally concentrated at a moderate frequency among teachers. In a study conducted in Sweden, 50.6% of participants obtained health-related information from the internet less than once a month (18). In studies conducted in China and Jordan, the rates of participants searching for health information daily were reported as 38.9% and 19.3%, respectively, while those who almost never searched were 9.4% and 5.1%, respectively (19, 20). The frequency of obtaining health-related information online varied across studies. These differences may be attributed to factors such as access to health systems, internet/social media use culture, burden of chronic diseases, and variations in how “search frequency” was measured in studies.

The most frequently used digital sources of health information among participants were Google (56%), physicians (54.5%), the Ministry of Health (38.2%), and artificial intelligence programs (28%). In Sweden, participants reported Google (47.9%) and Health Consultation e-services (33.7%) as their primary digital sources (18). In Jordan, the most frequently used sources were physicians (55.5%) and medical websites (18.7%) (20). These findings indicate that in most samples, the main gateways to digital health information are Google and physicians.

Teachers with a personal or first-degree relative's chronic illness were found to search for health information online significantly more frequently than those without. In the literature, the direction of this relationship varies across samples. For example, a study in Spain reported that participants without chronic illnesses searched for health information online more frequently (21). In a study from the USA, individuals with intense medical histories were found to perform more online health-related searches (22). Another study found that caregivers of relatives with chronic diseases used technology more often as caregiving intensity increased (23). Variations in findings across studies may result from differences in information-seeking motivation, caregiving burden, or digital access.

The rate of using Google and forums as digital health information sources was significantly higher among teachers who had a chronic illness themselves or in a first-degree relative. A study conducted in Saudi Arabia among patients with chronic diabetes found that Google was the primary search engine used for health information (24). Another study found that doctors were the most common source among participants with chronic diseases (25). Since chronic illness (whether personal or familial) increases information needs, these groups are expected to more frequently turn to easily accessible online sources.

In our study, the mean DHLI score of participants was 2.59 ± 0.38, indicating a moderate level of digital health literacy among teachers. Among the DHLI subscales, Protecting Privacy had the lowest score (1.78 ± 0.72), and Adding Content had the highest score (3.15 ± 0.69). The scores for Information Searching, Evaluation of Reliability, and Determining Relevance were at moderate levels. In a study conducted in Portugal among preschool teachers using the DHL tool, digital health literacy was reported as moderate to moderately high. In both studies, teachers appeared relatively competent in information searching, evaluating reliability, and determining relevance, whereas skills related to risk management and secure use in digital environments were limited (26). A study in Germany found that 60.7% of teachers had sufficient DHL. Similar to our findings, the lowest scores were reported in the Protecting Privacy subscale (27). A study in Iran assessing e-health literacy among teachers reported insufficient levels (28). In another Turkish study involving 1,274 participants (775 university students, 175 university graduates), the mean DHLI score was 3.04 ± 0.5, and the Determining Relevance and Navigation subscales scored moderately while others were sufficient (15). Variations in overall DHLI scores in the literature may be due to structural and contextual factors such as educational background, professional distribution, access to digital infrastructure, and national digitalization policies.

In this study, the mean DHLI scores did not significantly differ by age group, gender, marital status, or professional experience. The literature presents inconsistent findings on the relationships between sociodemographic variables and digital/e-health literacy. A study in Germany reported that younger teachers had better navigation skills, and women scored higher in information searching and relevance (27). In Taiwan, individuals aged 42–51 had lower functional e-health literacy than younger groups (29), while in Italy, men had higher DHLI scores than women (30). No significant relationship between gender and DHLI scores was found in China (19), and e-health literacy levels were similar across marital status in Jordan (20). A meta-analysis examining sociodemographic determinants concluded that gender and marital status generally had no significant effect, while age was negatively associated with e-health literacy in most studies (31). The lack of difference in DHLI scores by sociodemographic factors in our study is consistent with these variable/inconsistent findings. The role of professional experience in digital health literacy appears to be underexplored.

Only the Protecting Privacy subscale showed a statistically significant difference in favor of the group with 1–3 h of daily internet/social media use compared to those with 0–1 h. No significant difference was found between DHLI scores and frequency of online health information searches. Studies in Iran, China, and Sweden reported increased DHL levels with more frequent health-related internet use (18, 19, 28). Unlike these studies, our findings indicated that while frequency of searching did not affect DHL, overall internet use duration did. This difference may stem from variations in access to and trust in health services, internet/social media habits, and the measurement of “search frequency” across countries.

No significant differences were found in DHLI scores based on whether participants had experienced a serious diagnosis/treatment or had chronic illness themselves or in a close relative. Studies from Jordan and the USA also found no significant differences in e-health literacy levels based on chronic illness (20, 32). A review study reported a positive association between e-health literacy and chronic disease (33). The relationship may vary depending on context, country, and sample characteristics.

The mean scores for the Infodemic Scale were as follows: Awareness 40.24 ± 4.03, Literacy 22.12 ± 2.91, and Total 62.36 ± 6.35. These indicate high levels of awareness and literacy regarding infodemic. The proximity of these scores to the scale's upper limit suggests a potential ceiling effect, reflecting generally high levels of infodemic awareness and literacy among teachers. When most participants cluster at the higher end of the scale, it becomes more difficult to detect real differences between groups. This limited variation may also have reduced the ability to observe stronger statistical associations in the regression analyses (34).

The findings of this study should be interpreted in light of the regional context in which the data were collected. Gaziantep is a large city with relatively good access to digital technologies and health information (35). These conditions may shape teachers' online behaviors, trust in information sources, and exposure to health-related content. Therefore, the results may differ in other regions, particularly in rural areas or in places with more limited digital infrastructure. For this reason, the findings should be generalized to other contexts with caution.

No significant differences were found in Infodemic Scale scores based on age, gender, marital status, years of experience, serious diagnosis/treatment in the past year, or presence of chronic illness. The literature revealed limited studies using the same or similar scales to the Infodemic Scale, making it difficult to compare findings at the parameter level. A study by Geysynsky et al. examining perceptions of health misinformation on social media found no significant relationship between age or gender and perceived misinformation levels.

Teachers who used the internet/social media for 0–1 h daily had significantly higher Infodemic scores. This may indicate that this group, although less exposed to online content, approaches information more cautiously (36, 37), tends to rely more on official sources, and is more inclined to verify information before sharing.

Teachers who never searched for health information online had significantly higher Awareness subscale scores of the Infodemic Scale. This may reflect their perception of digital platforms as high-risk for misinformation (37), leading them to consciously avoid them and rely on official/expert sources.

Teachers who used TV as a digital health information source had significantly higher Infodemic scores. An international study found that greater exposure to traditional media (TV, radio, newspapers) was associated with lower levels of conspiracy beliefs and misinformation (38). A U.S. study predicted that individuals who used TV as an information source during the pandemic were significantly less likely to believe misinformation about COVID-19 vaccines, bioterrorism, and modes of transmission (39). These findings are consistent with our study; however, the heterogeneity of TV content and quality should be considered in interpreting the results.

Correlation analysis of the scales and subscales revealed strong positive relationships between awareness, literacy, and total infodemic scores. This suggests that although conceptually distinct, these subscales overlap significantly in measurement and may reflect the same overarching construct.

Simple linear regression showed that digital health literacy significantly and positively predicted infodemic scores, though the explained variance was low. This indicates that as digital health literacy increases, so does the infodemic score.

Among the subscales, Adding Content was the only significant predictor of infodemic level. This finding may indicate that individuals who actively produce or contribute content in digital environments are more likely to engage in verification, source evaluation, and responsible information sharing. In this sense, content creation appears to reflect not only a technical skill, but also a form of active digital participation and professional responsibility, particularly for teachers who play a role in disseminating information.

### 4.1 Strengths

This study provides data on a key professional group with a strategic role in information dissemination by jointly examining digital health literacy and infodemic awareness/literacy in preschool teachers working in public schools in Gaziantep, one of Türkiye's largest cities. The use of a multidimensional tool such as DHLI and the separate reporting of subscales enabled a clearer identification of strengths and areas for improvement. Reporting of scale reliability and demonstration through regression that digital health literacy significantly predicts infodemic scores strengthens the explanatory power of the findings. Identifying Adding Content as the only significant predictor in multivariate analysis also points to a practical, targetable skill area. Quantitative evaluation of a critical and current concept such as infodemic using a validated scale, along with analysis in relation to DHL, provides a more comprehensive picture of teachers' roles in the digital information ecosystem.

### 4.2 Limitations

The cross-sectional design limits causal interpretation. The sample being limited to one province may reduce generalizability to all preschool teachers in Türkiye. Although the predominance of female teachers limits gender comparisons, this reflects the natural composition of the profession (40). The use of self-reported online data may introduce measurement bias. Scores being close to the upper limit of the Infodemic Scale may have made it harder to detect real differences between groups due to a ceiling effect. Furthermore, the limited number of studies on infodemic in similar contexts and samples makes comparative discussion difficult. Lastly, although digital health literacy significantly predicted infodemic, the effect size was small, indicating that other individual/environmental determinants (e.g., media exposure, institutional trust, critical thinking, information verification habits) should be considered in future models.

## 5 Conclusion

This study demonstrated that preschool teachers have a moderate level of digital health literacy, with marked differences among subscales; particularly, while the “Adding Content” skill was relatively high, the “Protecting Privacy” skill was low-an important point in terms of digital risk management. Inter-scale analyses showed that digital health literacy positively and significantly predicted infodemic scores, though the explained variance was limited. The finding that “Adding Content” was the only subscale significantly predicting infodemic in the multiple regression analysis suggests that teachers' capacity not only to consume but also to produce/contribute digital content may be a critical competency in combating infodemic. Based on these findings, it is recommended that in-service training for preschool teachers should not only focus on skills such as accessing and verifying reliable information, but also target areas of digital privacy, data security, and competencies for appropriately producing/sharing evidence-based health messages in digital environments. Digital health literacy programs developed in collaboration with primary healthcare services and targeting teachers—as “key groups” in the dissemination of information within society-may contribute to strengthening infodemic management and public health interventions.

## Data Availability

The raw data supporting the conclusions of this article will be made available by the authors, without undue reservation.
